# Correction to: Eculizumab: A Review in Generalized Myasthenia Gravis

**DOI:** 10.1007/s40265-018-0889-3

**Published:** 2018-03-09

**Authors:** Sohita Dhillon

**Affiliations:** Springer, Private Bag 65901, Mairangi Bay, Auckland, 0754 New Zealand

## Correction to: Drugs 78(3):367–376 (2018) 10.1007/s40265-018-0875-9

The article Eculizumab: A Review in Generalized Myasthenia Gravis, written by Sohita Dhillon, was originally published Online First without open access. After publication in volume 78, issue 3, pages 367–376 Alexion Pharmaceuticals Inc. requested that the article be Open Choice to make the article an open access publication. Post-publication open access was funded by Alexion Pharmaceuticals Inc. Further details may be found at http://www.medengine.com/Redeem/39FCF0602D6721C2. The article is forthwith distributed under the terms of the Creative Commons Attribution-NonCommercial 4.0 International License (http://creativecommons.org/licenses/by-nc/4.0/), which permits any noncommercial use, duplication, adaptation, distribution and reproduction in any medium or format, as long as you give appropriate credit to the original author(s) and the source, provide a link to the Creative Commons license and indicate if changes were made.

